# Implementing a rapid fetal exome sequencing service: What do parents and health professionals think?

**DOI:** 10.1002/pd.6140

**Published:** 2022-04-14

**Authors:** Rhiannon Mellis, Dagmar Tapon, Nora Shannon, Esther Dempsey, Pranav Pandya, Lyn S. Chitty, Melissa Hill

**Affiliations:** ^1^ North Thames Genomic Laboratory Hub Great Ormond Street Hospital for Children NHS Foundation Trust London UK; ^2^ Genetics and Genomic Medicine UCL Great Ormond Street Institute of Child Health London UK; ^3^ Queen Charlotte's & Chelsea Hospital Imperial College Healthcare NHS Trust London UK; ^4^ Clinical Genetics Service Nottingham City Hospital Nottingham UK; ^5^ South West Thames Regional Genetics Service London UK; ^6^ School of Biological and Molecular Sciences St George's University of London London UK; ^7^ Elizabeth Garrett Anderson Institute for Women's Health University College London London UK; ^8^ Fetal Medicine Unit University College London Hospitals London UK

## Abstract

**Objectives:**

Prenatal exome sequencing (pES) for the diagnosis of fetal abnormalities is being introduced more widely in clinical practice. Here we explore parents' and professionals' views and experiences of pES, to identify perceived benefits, concerns, and support needs.

**Methods:**

Semi‐structured interviews were conducted with 11 parents and 20 health professionals (fetal medicine and clinical genetics) with experience of rapid pES prior to implementation in the English National Health Service. Interviews were transcribed verbatim and analysed thematically.

**Results:**

Parents and professionals were largely positive about pES, emphasising clinical and psychosocial benefits of a timely, definitive diagnosis in pregnancy. Concerns included parental anxiety related to the timing of pES results or uncertain findings, a need for guidelines for case selection and reporting, and ensuring sufficient capacity for counselling, phenotyping and variant interpretation. Professionals were concerned non‐genetics professionals may not be equipped to counsel parents on the complexities of pES.

**Conclusion:**

These findings highlight important issues for clinical implementation of pES. Expert counselling is required to enable parents to make informed decisions during a stressful time. To achieve this, professionals need further education and training, and fetal medicine and genetics services must work closely together to ensure parental understanding and appropriate support.

## Key points

What's already known about this topic?

- Prenatal exome sequencing (pES) increases genetic diagnoses in structurally abnormal fetuses.
- Health professionals (HPs) anticipate clinical utility from pES and recognise the need for expert counselling, informed consent and clinical guidelines.

What does this study add?

- UK HPs delivering rapid pES felt positive about pES but worried about variants of uncertain significance and incidental findings (IFs).
- Parents welcomed the offer of pES, provided they had sufficient time, support and opportunities to ask questions, both for decision making and after receiving results.
- Recommendations for successful clinical implementation of pES based on parent and HP feedback include improved multidisciplinary working, increased parental support and HP education.

## 1 INTRODUCTION

Prenatal exome sequencing (pES) increases the yield of genetic diagnoses in fetuses with ultrasound‐detected structural abnormalities where chromosomal microarray (CMA) is uninformative.^1^, ^2^, ^3^ Evidence to support the clinical utility of pES is growing and there are now guidelines from professional bodies that consider its use.^4^, ^5^, ^6^ Increasingly, pES is being adopted in clinical settings,^7^, ^8^, ^9^, ^10^ including the National Health Service (NHS) Genomic Medicine Service (GMS) in England.^11^ Alongside potential benefits for families, the use of pES raises practical, ethical and logistical considerations for its implementation in mainstream clinical practice.^12^, ^13^ These include ensuring a sufficient workforce with the necessary expertise, adequate laboratory infrastructure, education and training for professionals, clear guidelines, and support for families undergoing testing. Prenatal ES may also uncover genetic variants of uncertain significance (VUS) and incidental findings (IFs). These add to the complexity of pre‐test genetic counselling and conveying results, posing a challenge for parental informed consent, and generating anxiety for parents and professionals alike.

A growing body of literature has investigated parent^14^, ^15^, ^16^, ^17^, ^18^, ^19^ and HP^20^, ^21^, ^22^, ^23^ views and attitudes towards pES, which may guide implementation. However, few have explored direct experiences of pES in clinical practice, where results are returned during an ongoing pregnancy.^14^

Here we aimed to (1) identify perceived benefits of pES and anticipated challenges for its clinical implementation, (2) better understand the support and information needs of parents, and (3) consider the educational and training needs of HPs, by exploring stakeholder attitudes in a setting where results were returned in a timeframe to influence pregnancy management.

## 2 METHODS

Ethical approval was given by the London, Camberwell St Giles Research Ethics Committee (ref: 18/LO/0984).

### 2.1 Study design

This was a qualitative study using semi‐structured interviews to explore parents' and HPs' views and experiences of pES.

## 3 SETTING

Participants were recruited from five NHS hospitals offering rapid pES: four in London and one in the East Midlands. Four hospitals offered pES within a research study using pES to diagnose suspected skeletal dysplasias, with analysis targeted to a virtual panel of 240 skeletal dysplasia genes.^24^ One also offered pES on a research basis for isolated cardiac anomalies, analysed using a panel of ∼1000 genes associated with fetal structural anomalies. The fifth hospital offered pES in a clinical setting for fetal structural abnormalities, with analysis targeted to patient‐specific gene panels relevant to the fetal phenotype in each case.^25^ Results were returned to parents in an average of 3 weeks. Secondary findings were not looked for, but IFs with implications for a family's health were reported. VUS were reported in exceptional circumstances, for example when found in trans with a (likely) pathogenic variant in a recessive gene relevant to the fetal phenotype.

### 3.1 Participant recruitment

Parents offered pES, aged over 18 years and able to communicate in English, were identified by the local clinical teams and invited to complete an interview about their experience 3–18 months after receiving results. Participant information was posted with a request to contact the study team with any questions or if interested in taking part. If there was no response after 2 weeks, a local clinician telephoned potential participants to discuss the study. Parents were offered a £10 gift voucher in appreciation of their time. Fetal medicine and clinical genetics HPs involved in delivering rapid pES were identified by the study team and emailed the participant information, with a request to contact the study team with questions or to take part. Written or verbal consent was obtained from each participant prior to the interview.

### 3.2 Interviews

Interviews were conducted by MH and RM, face‐to‐face or by telephone. Topic guides were initially drafted by MH, an experienced qualitative researcher, and revised following feedback from LSC and RM, clinicians with experiential knowledge of pES. Topic guides included: (1) Experiences with pES; (2) Perceived benefits and limitations; (3) Pre‐and post‐test counselling; (4) Results of pES and their impact; (5) Views on clinical implementation; and (6; for HPs only) Service delivery and training needs for HPs. (See supporting information for topic guides). Demographic information was also collected (Tables S1 and S2).

### 3.3 Data analysis

Interviews were digitally recorded, transcribed verbatim and anonymised. Data were analysed using thematic analysis centred around a codebook.^26^ Parent and HP interviews were treated as a single dataset. A draft codebook was initially developed by MH, based on the topic guides and existing literature. Using this, MH and RM independently coded the same two HP transcripts and added further codes, discussing any discrepancies and agreeing a revised codebook to guide coding of the remaining HP transcripts. This process was repeated for the parent transcripts. Codes were reviewed and revised throughout the analysis, with additional codes added when identified inductively from the data. All transcripts were coded by RM, with six HP and four parent interviews coded independently by MH to check agreement between researchers. Codes were grouped to identify themes and sub‐themes within the data. NVivo version 12 software (QSR International, Pty Ltd, Australia) was used to facilitate organisation and coding of transcripts.

## 4 RESULTS

### 4.1 Participants

Of 26 HPs invited to participate, six did not respond and 20, from a range of roles (Table S1), were interviewed (recruitment rate: 77%) between September 2018 and February 2020. Interviews (10 face‐to‐face and 10 by telephone) lasted between 14 and 52 min (median 32 min).

Of 30 parents invited to participate, 13 did not respond, three actively declined and 14 parents from 11 families were interviewed (recruitment rate 47%). Two transcripts were excluded from the dataset: one couple consented for pES but received a diagnosis from single gene testing first, and one parent was offered exome sequencing (ES) after termination of pregnancy. Therefore, the final parent dataset comprises 11 parents from nine families. Interviews were conducted between March 2019 and December 2021 (2 face‐to‐face and 10 by telephone) and lasted between 33 and 63 min (median 43.5 min). Parents had a range of experiences with pES and pregnancy outcomes (Table S2).

### 4.2 Overarching themes

Views on pES fell into four overarching themes: (1) Positive perceptions of pES, (2) Potential for negative impacts on parents, (3) Challenges for supporting parents when pES is offered, and (4) Challenges for service delivery. Any topics or themes emerging from only one participant group are noted.

### 4.3 Positive perceptions of prenatal exome sequencing

#### 4.3.1 Professionals and parents embrace prenatal exome sequencing in clinical practice

Parents and HPs generally felt positive about implementing pES in clinical practice, describing it as “amazing”, “exciting” and “promising” (Table 1, Q1). Parents welcomed the offer of pES and were motivated to take up testing to gain as much information as possible about the cause and prognosis of their baby's problems (Table 1, Q2), and to identify recurrence risks for future pregnancies.

**TABLE 1:** Positive perceptions of prenatal exome sequencing (pES)

Topic areas and representative quotes
Professionals and parents embrace pES in clinical practice
Q1: “*I think it's something that you guys should offer to everybody in my situation, because it will help them the same way it helped me.”* P5, mother with diagnostic findings from pES (ToP before result)
Q2: *“We were very, very keen on [the offer of pES] because if we can have all the information and we have some detail on the specific disease, that would be great because we'll have a clear idea of, or a clearer idea of what's going to happen or what kind of quality of life the child would have because, you know, we would look at past cases and see how those children's lives have panned out*.” P14, father with probable diagnostic finding from pES (ToP before result)
Q3: *“I think there are obviously a few challenges like the turnaround time, who will inform patients of the results, who will do the consenting, but thinking back to all the changes that I've seen moving to new testing, I think we always managed it and really I don't – I can't see that many different new issues that we didn't have before. I mean, I remember we had exactly the same discussions when array was started and now it's a standard test.”* HP1, genetic counsellor

Clinical benefits
Q4: *“You'*re *going to have a higher chance of making a diagnosis, a definite diagnosis for that family than you would have by conventional means.”* HP4, clinical geneticist
Q5: *“It was bad news, but we were grateful that we had the results because now we know what our chances are… So, yeah, I was very surprised when I found out that I had chosen a partner who had the same mutation in the same gene, yeah, and I was upset that it had gone down to 1 in 4 chance, but I was still grateful to have found out. Because it just makes you kind of plan, if that's possible when trying to have children…it's just better to know so that you can try and plan as best you can.”* P11, mother with probable diagnostic finding from pES (ToP before result)
Q6: *“Well, tests and screening tests are very time critical in pregnancy, thinking about if women decide they want to interrupt the pregnancy and just getting an answer as soon as possible I think would be of enormous value to families and women if they think they might want to end the pregnancy”* HP15, fetal medicine midwife
Q7: *“We had another case where we wondered if the baby had osteogenesis imperfecta. When we analysed the variants we found that the baby in fact had hypophosphatasia. As there is now an enzyme replacement for hypophosphatasia we were able to set it up prenatally for immediate postnatal treatment with our colleagues, and she was sent to [a specialist hospital] for treatment as soon as the baby was born, and the baby's doing very well.”* HP18, clinical geneticist
Q8: *“I think also knowing the diagnosis antenatally, means that that neonate isn't going to go through a barrage of investigations that are both physically expensive – they'*re *time consuming, they'*re *mentally expensive for parents and just horrific, that kind of diagnostic odyssey that babies with metabolic disorders go through before they get a diagnosis. And if we know that before they'*re *born, maybe we* save *them a bit of that.”* HP20, clinical geneticist

Psychosocial benefits for parents
Q9: *“That was incredibly important for me to have that information because then that helped me then have solid information from which I could then base decisions on and research and understand what it was that we were dealing with.”* P9, mother with diagnostic finding from pES (Live birth)
Q10: *“Knowledge is power as I said before, you know, but yeah, anything that can give parents hope and feel – and a bit of control over the situation as well, you know, has got to be helpful.”* P12, mother with a diagnostic finding from pES (ToP before result)
Q11: *“I mean some of the upset is because they don't know what's wrong with the baby. So I do believe that we are helping by doing these tests, by doing these investigations, to take away the uncertainty if we can.”* HP18, clinical geneticist
Q12: *“I think it's fantastic because I think if I'd gone through it not knowing what the problem was that it would have been a thousand times more distressing”* P10, mother with diagnostic finding from pES (ToP after result)
Q13: *“So that was reassuring as well, once we had the results, to find out there was nothing actually from us, that it was something just to do with that particular baby…and obviously that was always going to be nice to know, for the next baby.”* P5, mother with diagnostic finding from pES (ToP before result)
Q14: *“It was reassuring in a way, obviously that's a way of thinking she's going to be OK most of the time. But we know there is a problem, definitely, but at least some [conditions] we can actually rule out”* P2, father with no diagnosis from pES (Live birth)
Q15: *“Knowing that there was also something else that was going to affect and limit his life, you know, the poor kid who would have had not only an undesirable heart, let's say, but also the other issues he would have faced because he was suffering with [a genetic syndrome], so those two combined, it made us feel 100%, you know, we don't doubt our decision…so knowing that we really had done the best thing was again a huge comfort.”* P12, mother with diagnostic finding from pES (ToP before result)

HPs' enthusiasm for the routine implementation of pES was tempered by comments around potential practical and ethical challenges. Several noted, however, that many similar challenges were already addressed when introducing prenatal CMA into routine practice (Table 1, Q3).

#### 4.3.2 Clinical benefits

Both groups valued the improved diagnostic yield of pES (Table 1, Q4). Other clinical benefits discussed included better counselling about prognosis, guiding pregnancy and perinatal management, defining recurrence risk, and providing options for prenatal diagnosis in future pregnancies. For example, one couple accessed early targeted non‐invasive prenatal diagnosis in a subsequent pregnancy, and another described how knowing they had a one in 4 recurrence risk informed their reproductive planning (Table 1, Q5). Health professionals highlighted that interrogating many genes in a single test enables faster diagnosis, to inform management during an ongoing pregnancy (Table 1, Q6). Clinicians gave examples of rapid diagnoses from pES enabling early access to specialist services or treatments (Table 1, Q7) or planning for palliative care, as well as circumventing numerous painful and time‐consuming post‐natal investigations (Table 1, Q8).

#### 4.3.3 Psychosocial benefits for parents

Parents and HPs reflected on psychosocial benefits for parents: enabling them to *“make decisions with a better information base”,* giving them the option to contact relevant support groups, helping them to be prepared practically, and giving them a “*little bit of time to recover emotionally*” before the birth (Table 1, Q9). Parents took comfort in doing something proactive at a *“very scary time”* (Table 1, Q10) and both groups frequently discussed the alleviation of uncertainty through diagnosis (Table 1, Q11‐12). One HP spoke of a couple's “*enormous relief that they could know what they were up against”*. Parents who received a diagnosis also described relief *“that actually there was nothing [they] could have done”* and reassurance that *“it wasn't [their] fault”.* Findings where the molecular diagnosis carried a better prognosis or the result showed a de novo variant with a very low recurrence risk in future pregnancies were also reassuring to parents (Table 1, Q13). Parents with a ‘no findings’ result from pES could also feel reassured that some conditions had been ruled out (Table 1, Q14).

Four of the nine families interviewed had decided to end a pregnancy based on scan findings before receiving their pES result and all noted that a confirmed genetic diagnosis gave them *“some kind of comfort”,* reassured them, and allowed them to feel more confident that they *“made the right decision”* (Table 1, Q15). One parent who opted for a surgical termination of pregnancy was grateful that pES provided diagnostic information that avoided the need for a post‐mortem that would have required medical termination and delivering the fetus, which she felt might leave her “*traumatised*”.

### 4.4 Potential for negative impacts on parents

#### 4.4.1 Generating further anxiety

Both groups discussed the potential for pES to exacerbate parents' anxiety around the health of their unborn baby, particularly since results from pES take longer than traditional testing, thereby prolonging the period of anxiety (Table 2, Q1). Whilst technically 3 weeks can be considered a ‘rapid’ turnaround time for pES, for parents this can be an unbearably long wait (Table 2, Q2). Additionally, some parents described the emotional ups‐and‐downs resulting from multiple sequential tests whereby *“shock”* from the scan findings could shift to *“relief”* following no findings from a karyotype or microarray and then crash again with pES findings (Table 2, Q3).

**TABLE 2:** Potential for negative impacts on parents

Topic areas and representative quotes
Generating further anxiety
Q1: *“It's always a really difficult time for people to try and make these decisions, and this could extend that period of uncertainty as well.”* HP9, genetic counsellor
Q2: *“We were told that it was going to be – was it 3 weeks wait or, like, quite a long wait to find out the results – so you'*re *sat there 20 weeks' pregnant, you'*re *aware that you can't let – if I'm going to do something I need to do this sooner rather than later because I can't emotionally have a baby growing inside me…”* P12, mother with diagnostic finding from pES (ToP before result)
Q3: *“so they explained a bit more about what the high nuchal translucency might mean and they were obviously firstly looking for Down's, Edward's Patau's… so the chorionic villus sampling results [from karyotype] came back really quite quickly and we sort of felt this kind of relief! And then they said they'd also look for any kind of bits that were duplicated or missing – and that had come back clear – so we felt completely relieved… Then we went back for another scan and it hadn't improved, it had got worse… and they said that they thought it was mostly likely to be [a monogenic condition] and explained that they could use the same samples from us [for pES].”* P10, mother with diagnostic finding from pES (ToP after result)
Q4: *“So in my experience, if you have a diagnosis, it makes it much easier to decide what to do about the pregnancy, whether you continue or whether you stop the pregnancy. But the patients, the couples that find it most difficult are the ones where there is uncertainty.”* HP18, clinical geneticist
Q5: *“But for things such as unknown significance results or something that may have some implications that are relatively mild, the difficulty would be that if that result is given after 23 weeks and two or 3 days, that couple may not have enough time to come to a decision and be offered termination of pregnancy…* w*e'*re *meant to offer people choice, but if in the end we cannot offer them choice and we just offer them extra worry, it's not going to be helpful.”* HP16, fetal medicine consultant
Q6: *“I was up against like a ticking bomb, to be honest, because I had to do the termination before 24 weeks”* P11, mother with a probable diagnostic finding from pES (ToP before result)
Q7: *“It was very, very difficult, we were talking about it, ‘but what if it's not the lethal kind?’, ‘are we doing the right thing?’ But in terms of the exome sequencing, I think if we had had those results prior to 24 weeks – and the reason that we didn't [wait] was because of the lab closure – I think that would have really, really put a lot – you know, it was a really difficult time and I think that would have made that difficult time slightly less difficult.”* P14 (partner of P11), father with a probable diagnostic finding from pES (ToP before result)

Both positive and negative results can be disappointing
Q8: *“Negative results can be very helpful and can be reassuring. They can also be disappointing when there's a clear, clear problem.”* HP20, clinical geneticist
Q9: *“you know, it was kind of, like, this has gotten worse, you know, it went from just being a De Novo 1 in 70,000 now gone into 1 in 4.”* P11, mother with diagnostic finding from pES (ToP before result)

Incidental findings may create an additional worry
Q10: *“And it is a bit of a worry that if these sorts of things are being picked up, particularly at a time when someone's pregnant – if there's abnormalities on a scan and they'*re *already trying to decide what to do – if these sorts of things are coming up as well, it could be very, very difficult for somebody to try and deal with this extra news at that time.”* HP9, genetic counsellor
Q11: *“I suppose we were just a bit worried that if we agreed to the exome testing, that other things might be revealed about ourselves…You know, a condition, not necessarily that would affect another baby – so I wasn't really even thinking about that, it was more about discovering we've got a gene that's gonna mean I've got some terrible disease or whatever.”* P6, mother with diagnostic finding from pES (ToP before result)

Several HPs felt that uncertainty arising from a VUS or a no‐findings result was a major source of anxiety and the most difficult situation for parents to deal with (Table 2, Q4). The timing of pES results in relation to legal limits for pregnancy termination was identified as a potential source of additional stress, as after 24 weeks gestation UK law permits termination only where there is ‘substantial risk’ of serious disability. If the pES result is uncertain or does not meet this criteria, parents may no longer have the option of a termination (Table 2, Q5). One couple described the pressure they felt to make a decision before the 24 weeks *“deadline”* when their pES result was delayed (Table 2, Q6‐7).

#### 4.4.2 Both positive and negative results can be disappointing

Health professionals observed that any result can have negative impacts for parents, including disappointment with a ‘no findings’ result (Table 2, Q8) and the possibility that a diagnosis *“takes away their hope that there's nothing wrong”*. One parent described their ‘no findings’ result as *“reassuring in a way but obviously it's worrying in another way as well because they could not find [a diagnosis]”* (P2, Father with no diagnosis from pES). One who received a diagnosis with a poor prognosis described their result as “*pretty devastating*”, and another couple were shocked to learn they were both carriers for the condition that affected their pregnancy and therefore had a 25% recurrence risk (Table 2, Q9).

#### 4.4.3 Incidental findings may create an additional worry

One genetic counsellor voiced concern that returning IFs with implications for parents' own health, such as a cancer predisposition, may present an additional worry at an already difficult time (Table 2, Q10). For one couple, this risk of IFs was the reason they initially had reservations about pES (Table 2, Q11).

### 4.5 Challenges for supporting parents when prenatal exome sequencing is offered

#### 4.5.1 Interpreting and communicating uncertain and complex information

Prenatal ES has the potential to detect more IFs and VUS than previous testing methods – *“the more we look, the more we will find”* – and HPs felt these concepts are particularly challenging to convey to parents (Table 3, Q1). Some emphasised limitations in pES pipelines and our ability to interpret exome data that need to be described to parents (Table 3, Q2), and others highlighted challenges with incomplete fetal phenotyping, which may evolve so that variant classification may even change when revisited postnatally (Table 3, Q3). Thus, discussions about pES were perceived as more complex than other prenatal genetic tests and HPs recognised that they need a thorough understanding of the possible outcomes and limitations of the test before they can communicate these to parents (Table 3, Q4). Parents reported that unfamiliar medical terminology was difficult to grasp but nonetheless demonstrated accurate and often nuanced understanding of the goals and limitations of pES and of the results they received, including a VUS with the potential to be reclassified and appreciating that a ‘no findings’ result does not exclude a genetic condition (Table 3, Q5).

**TABLE 3:** Challenges for supporting parents when prenatal ES is offered

Topic areas and representative quotes
Interpreting and communicating uncertain and complex information
Q1: *“the concern is that whole exome sequencing will highlight – will give us results that we find difficult to interpret, and difficult to communicate to the people that we'*re *looking after, that don't necessarily give the certainty that we would like them to give.”* HP13, fetal medicine consultant
Q2: *“I think it's very, very important that the patients understand the limitations of these tests because the sort of pipeline that we have is that we do a virtual panel, we do not look at everything… So I think parents, when you say that we've got this new technology and it's amazing and we can test all these genes all at once, they have the potential to think that we are testing everything, and that the absence of an exome diagnosis means a normal baby, and obviously the onus is on us to make sure that they don't believe that we can ever test for normality.”* HP20, clinical geneticist
Q3: *“From our point of view the interpretation of variants can be more tricky in a prenatal [test]. We don't have the complete phenotype necessarily, because the scan is just a very limited, it's not like you've got a child [in front of you] so you can't see their intellectual disability and things like that. You can't get that information from a scan.”* HP11, genetic scientist
Q4: *“I think the importance is when you'*re *counselling women, the people who are doing the counselling are fully aware of the limitations of the testing and what it potentially can and can't tell families.”* HP15, fetal medicine midwife
Q5: *“So they did the research as I said, it all came back negative – Obviously the doctor said that's just a limited resource that they have available, it doesn't mean that there's not a problem, it's just they don't know what the problem is.”* P2, father with no diagnosis from pES (Live birth)

Supporting informed decision making
Q6: *“I suppose we spent those couple of days [awaiting specialist referral] kind of swinging from you know, having to face the facts that there might be something and then trying to hope for the best really. But yeah I mean it was not a good time at all.”* P6, mother with diagnostic finding from pES (ToP before result)
Q7: *“And it's very difficult to fully consent people before performing that kind of testing. I mean we do our best, but truly do people understand what information might be gained from these tests? I think that's a very difficult thing to communicate.”* HP13, fetal medicine consultant
Q8: *“So I think – although it's always difficult isn't it, when I think – when patients particularly are pregnant, is that feeling – that pressure from them that they would want to find an answer and they'*re *willing to do anything, which is what a lot of couples say.”* HP6, genetic counsellor
Q9: *“No [we didn't have any hesitation about accepting pES], I think it was offered and we were so hungry for having any type of information about what was the matter with him that we were willing to do it.*” P9, mother with diagnostic finding from pES (Live birth)
Q10:*“at this point we'*re *so relieved that there is not a really bad abnormality with the heart, the kidney is manageable. We absolutely want to know if all of these forty six chromosomes look normal but to do further testing to see if there's some sort of like, something that might affect the growth of the child's bones, but isn't really directly linked to a really serious disability or illness, or that she might have a learning difficulty, didn't really seem like we wanted to open Pandora's box and go into that rabbit hole.”* P8, mother who declined pES (Live birth)
Q11: *“I think the majority are perfectly capable of deciding and taking informed decisions and I think we shouldn't be too paternalistic and think this is too complicated for them or they wouldn't understand or they would just worry – I think people tend to worry a lot more when they don't have information”* HP1, genetic counsellor

Time, written information, and emotional support are needed
Q12: *“[The genetic counsellor] was just so good, and she was always there if I needed to call her. I could always get hold of her. She'd always call me back. Loads of times, it was actually her – she was really proactive. She would call me, and say ‘hi how are you doing?’ It was just nice to have that kind of support really. And it always gave me an opportunity to ask a question if I had one.”* P5, mother with diagnostic finding from pES (ToP before result)
Q13: *“Maybe that's something that should just be followed through anyway, that actually 2 days later somebody should have called me because after everything that we did on that day, and so much information to take in, it would have probably helped if they'd had someone just check in and say you know, are you OK, and are you happy that we'*re *still going to go ahead with this. Because they probably did say “if you've got any questions give us a call” – but you know, people don't do they?...You need someone else to take the lead on it.”* P6, mother with diagnostic finding on pES (ToP before result)
Q14: *“[We should not] forget about the emotions and psychological stress on the parents…We quite often forget about this, and we just go around our business and we say “OK, you'll have this test or other, and you can have this test or other”. And we forget that these patients are actually wrestling with the emotions at the time. So during the – any process of consent for any test, especially an invasive test, or this kind of deep searching in the human genome, we have to remember about the emotions on the other side and not just treat it lightly.”* HP17, fetal medicine doctor
Q15: *“I think it's [important] just to develop a bit more of a holistic humanitarian approach to, like, help support families through accepting the diagnosis that they receive and that just keeping top of mind that yes this is great that you'*re *getting more information but then how do you impart the information and how do you make sure that people are supported enough processing that information.”* P9, mother with diagnostic finding from pES (Live birth)

Offering ‘looked for’ additional findings
Q16: *“I personally don't feel very comfortable with looking for secondary findings in a prenatal context. I think the reason that the test is being requested is usually because there are abnormalities that are being seen on scan and that's what you'*re *looking for the explanation for and you'*re *trying to help couples make decisions in quite a short timeframe and I think starting to look at things outside of that, that are unrelated to that, I don't think that I would feel very comfortable about putting that extra burden on the family when it wasn't something that they'd been initially referred to us for.”* HP4, clinical geneticist
Q17: *“Well we didn't really want to know [any incidental findings], which is why we were a little bit hesitant. But I don't know, somebody else might really want that. Maybe perhaps if that's there as an option…[but] if you'*re *going to offer that, I think that is a really big decision that needs a lot of thinking about, and a lot of explaining?… So I think there's going to need to be a lot of thinking about how that's approached really, to make sure people really do have an informed choice.”* P6, mother with diagnostic finding from pES (ToP before result)

#### 4.5.2 Supporting informed decision making

Following detection of fetal anomalies, parents described feeling shocked and devastated, often followed by an “*emotional rollercoaster”* of anxiety, isolation, and uncertainty (Table 3, Q6). In such an emotionally charged and often time‐pressured context, some HPs worried about ensuring parents made informed decisions regarding pES (Table 3, Q7), particularly when parents are often *“desperate”* for more information and would *“do anything”* to find the answer (Table 3, Q8‐9).

Parents also commented that, in this stressful situation, they found it difficult to absorb information and often struggled to later recount specific details because *“it was all a bit of a blur”*. Several reported they *“didn't actually care about”* technical details, they *“just wanted to know what the answer was”*. The *“emotional rollercoaster”* also influenced parents' decision to decline pES. When offered pES, one couple were experiencing relief following news of an improved prognosis based on scan findings. After reaching that point of relative reassurance, they felt the offer of pES came suddenly and the new “*unknowns*” of what it might reveal introduced unwanted uncertainty (Table 3, Q10).

Overall, parents felt that they had enough information to decide about pES and HPs were confident that informed decision‐making was possible with appropriate support (Table 3, Q11). Importantly, no parents in our study expressed regret about their decision to accept or decline pES.

#### 4.5.3 Time, written information, and emotional support are needed

Parents and HPs viewed as essential pre‐test counselling that clearly and succinctly covers key points, manages expectations, explains timelines for testing and outlines potential results. Recommendations for counselling content were made by both groups (Table 4) with emphasis on parents needing “*a little bit of time to digest information”*, and multiple opportunities to ask questions. Parents appreciated active follow‐up from their clinical team to check their understanding after initial pre‐test counselling and suggested this as an area for improvement (Table 3, Q12‐13). The importance of attending to parents' emotional as well as informational needs was also highlighted (Table 3, Q14), including support after the return of results, whether continuing or ending a pregnancy (Table 3, Q15). Accessible information for parents to refer back to was considered important. Parents suggested that simplified language, a glossary of key terms, and ‘real life’ examples of possible test outcomes would improve current written information.

**TABLE 4:** Participants' recommendations for content to cover in pre‐test counselling for Prenatal exome sequencing (pES). Greyed out cells indicate where a point was raised by one group only

Described by health professionals	Described by parents
The pES result may provide an explanation for the fetal abnormalities, which could help with management, decisions in the pregnancy and information about recurrence risk for future pregnancies	Explanation of the potential advantages of a result to parents for their decision making
	Potential benefits for future pregnancies as well as current pregnancy
A basic explanation of genes and sequencing and how this provides higher resolution than chromosomal testing (further technical details of the test should be discussed on a case by case basis, depending on how much individual parents want to know)	Simple and basic explanation of genes, chromosomes and inheritance and how exome sequencing works, described in layman's terms
We might not find a genetic cause ‐ this could mean that there is no genetic cause for the fetal anomalies or it could mean that there is a genetic cause that pES is unable to detect	The fact that you may not find anything or may not get a definitive answer
Even if we do find a genetic diagnosis it might not tell us how severe the condition will be for this baby	
We might find an uncertain result (VUS) and be unable to interpret whether or not it is causing the fetal abnormalities	
We might find an incidental finding – a result which does not explain the fetal abnormalities but has other health implications for the fetus, parents or wider family	Explaining that you might find something unexpected and related to parents' own health but also being clear that this is unlikely because the test is focussing on genes associated with fetal abnormalities
We may detect a variant that we don't think is significant now, but as our knowledge improves, that may change later	
	Expected accuracy of the test
The test will reveal misattributed paternity if present	
Practical aspects of testing: How long it will take for results, who will inform parents of the results and how (e.g. face to face, telephone call or letter)	What tests are being done, in which order, and when to expect results of each.
Be clear on what types of results will or will not be looked for (e.g. if targeted gene panels are being used, or if incidental findings will not be reported etc.)	What is being tested and what are the possible outcomes – what you will and will not be able to find, including examples of the kinds of conditions that could be diagnosed
Explore with parents their motivations for testing, how they would cope with different results scenarios, and what their options would be, to help them decide whether they want the test	Discussion of what a result might mean for the baby and parents, including what will happen next if something is found
Discuss timing and explain that if the result comes back close to or beyond 24 weeks' gestation then the option of termination may not always be available	
	Keeping information simple and written information in accessible language that is easily understood
	Who to contact in case of further questions

#### 4.5.4 Offering ‘looked for’ additional findings

Health professionals largely did not support offering additional ‘looked for’ findings from pES but were uneasy about withholding such information if detected incidentally (Table 3, Q16). In contrast, parents held varied views on receiving additional findings with implications for their own health but were mostly in favour of being offered the choice to ‘opt in’ to receive these. It was noted that this would require additional explanation and careful consideration to ensure parents can make an informed choice (Table 3, Q17).

### 4.6 Perceived challenges for clinical service delivery from Health professionals

#### 4.6.1 Collaborative multidisciplinary team working will be essential

All HPs expressed their preference for pES to be led by genetics professionals, with some considering a transition to fetal medicine‐led delivery may be appropriate as experience grows (Table 5, Q1). MDTs were considered essential for effective service delivery and should involve clinical genetics, fetal medicine and clinical scientists to discuss differential diagnoses, determine suitability of pES for potential cases, and to interpret the significance of genetic variants (Table 5, Q2).

**TABLE 5:** Perceived challenges for clinical service delivery from Health professionals (HPs)

Topic areas and representative quotes
Collaborative multidisciplinary team (MDT) working will be essential
Q1: *“for now, I think it works well with these patients coming to genetics, because we've got that expertise. As this becomes more routine, as we get better at doing it, maybe it will just fit in, slot in better with the fetal care team.”* HP6, genetic counsellor
Q2: *“The question then is in the presence of a fetal anomaly should we be doing microarray or should we be doing exomes. I think that should be driven by a partnership between fetal medicine and genetics.”* HP14, fetal medicine consultant

Resources and infrastructure
Q3: *“Not everyone has a genetic counsellor in the unit or easy access, so are they all going to employ someone? I mean, it would be great…”* HP1, genetic counsellor
Q4: *“The only other thing is that this is creating more work for us which I'm happy to do because I believe in it and I think it's useful…but if it does become more frequent…we'*re *going to struggle to cope with that workload. So the clinical capacity needs to be considered.”* HP19, clinical geneticist
Q5: *“You'*re *going to need a tight turnaround because these – you don't want pregnant people waiting for a result. But along with the tight turnaround, you need staff to do the testing, and to do the analysis. So it's going to have quite a significant impact on the department.”* HP10, genetic scientist

Upskilling of the workforce
*Q6: “I don't think fetal medicine doctors are sufficiently experienced and knowledgeable at the moment and I speak as a – you know I think I'm reasonably up to date, but I don't think I am sufficiently expert to counsel.”* HP12, fetal medicine consultant
Q7: *“It's important to have some grasp, even if it's a basic grasp because if I'm not able to explain something fully but I can give some basic understanding I can always say ‘well look, I'm not the best person to speak to, a genetic counsellor would be the best person, however, this is essentially what it means’. So I think, yeah, I mean, that's the thing, it would be really beneficial to get some education that can be made available for everyone.”* HP3, fetal medicine midwife

National guidelines are needed
Q8: *“I think it's good if there's a diagnostic indicator to do it. I think it would be a terrible idea to just be offering it to everybody. So we definitely need good defined criteria for offering the test.”* HP10, genetic scientist
Q9: *“So I see that it will be phenotype based, and certainly as is always the case, there will be initially at least, real limitations from a funding perspective within the NHS, over you know, we won't be offering a really expensive test if the yield is likely to be very low. So we will have to use our resources wisely, and that will mean offering exome sequencing to pregnancies where we know the yield is going to be that much higher, I think.”* HP13, fetal medicine consultant
Q10: *“Personally I think its biggest role in the future is going to be for more subtle abnormalities. So I think actually that it may make you to have a lower pick up rate, but it would make a much bigger difference to the decision making for that couple. So I personally would like to see a lower threshold for doing fetal exomes.”* HP19, clinical geneticist
Q11: *“We've got no set of guidelines yet on what should be put in a report and that needs to be developed before we can start offering it on a diagnostic basis”* HP11, genetic scientist
Q12: *“If it's a variant of unknown significance that can be clarified by doing further testing perhaps in the family, then we should report it. And if it's not looking very likely and there's nothing else we can do, then my personal preference is not to report it. It creates a lot of anxiety and a lot of work, and if there's no way of resolving it, it doesn't seem a useful thing to do.”* HP19, clinical geneticist
Q13: *“I don't think you can ever say ‘this is the right way to do it or the wrong way to do it’. So as long as we all agree to a sensible way, that's fine, but we must all do the same because if somebody offers it to everybody and somebody offers it to specific findings then it gets more challenging.”* HP16, fetal medicine consultant

#### 4.6.2 Resources and infrastructure

Many HPs felt that genetics professionals have the best skill‐set to conduct pre‐test counselling conversations, but noted logistical and staff capacity challenges that made this difficult to achieve (Table 5, Q3). Concerns were also raised around the extra time needed for phenotyping and variant interpretation, over and above existing workloads (Table 5, Q4). Clinical scientists highlighted that more laboratory staff and efficient workflows will be needed to maintain fast turnaround times in the face of increased test uptake, as the analysis for each case can be time‐consuming (Table 5, Q5).

#### 4.6.3 Upskilling of the workforce

Health professionals noted that levels of awareness and experience of genomics and pES will vary widely amongst fetal medicine professionals, and that they may lack the necessary experience and training to confidently conduct pre‐test counselling, interpret and return complex or uncertain findings to patients (Table 5, Q6). Specialist training and genomics education was considered essential, tailored to the needs of each professional group. Basic awareness of pES may suffice for midwives supporting women receiving results, but any HP taking informed consent would require more detailed understanding of the test and its limitations (Table 5, Q7). One clinical geneticist also noted the importance of training geneticists in fetal medicine to support the clinical service. Seminars, self‐paced online modules, and ‘on the job’ exposure to pES were all valued as educational tools, with most HPs advocating a combination of learning formats.

#### 4.6.4 National guidelines are needed

Health professionals emphasised the need to define eligibility for pES testing, noting that evidence to show which pregnancies benefit most remained sparse (Table 5, Q8) and there was limited published guidance. Some HPs felt testing should focus where there is highest likelihood of a diagnosis, to maximise diagnostic yield with limited resources and simplify interpretation (Table 5, Q9), whereas others advocated a lower threshold for testing, as in some cases with milder or non‐specific phenotypes the diagnostic yield is lower but results may have an important impact on pregnancy management (Table 5, Q10).

Health professionals also wanted formal guidelines on reporting results, especially whether and when to report IFs or VUS (Table 5, Q11). VUS were frequently viewed as “*not helpful*” but HPs felt that they should be disclosed if likely to be clinically relevant (Table 5, Q12). Regardless of the content, guidelines should be consistent and nationally agreed, to ensure fair allocation of resources, and equity of access (Table 5, Q13). Some HPs anticipated challenges around regulating the private sector, concerned that pES might be offered without the same standards of counselling and interpretation expected within the NHS.

## 5 DISCUSSION

Consideration of all stakeholder views is key to delivering safe, effective pES that meets parent and HP needs. Our study differs from many others^15^, ^16^, ^17^, ^18^, ^19^, ^20^, ^21^, ^22^, ^23^ in that parents received results rapidly and so these could inform current pregnancy management as well as future reproductive choices. As with other studies,^14^ ^,^ ^15^ ^,^ ^17^, ^18^, ^19^ ^,^ ^21^, ^22^, ^23^ all participants saw clear benefits of pES providing a genetic diagnosis for more pregnancies with fetal anomalies, and many articulated associated positive clinical impacts (Table 1) similar to those described in clinical utility studies.^1^ ^,^ ^2^ ^,^ ^7^, ^8^, ^9^ ^,^ ^25^ ^,^ ^27^ Our findings mirror previously reported psychosocial benefits for parents, including being informed and prepared,^19^ feelings of relief from a negative result,^14^, ^15^ and gaining ‘closure’ from a diagnosis.^15^, ^19^ As noted in a recent study by Plantinga et al., we found that parents may opt to end a pregnancy without waiting for pES results but still valued a diagnosis for reinforcing the decision that was made.^14^

We observed that pES may have unintended negative impacts on parents, for example, prolonging anxiety while awaiting results and, like others, found that both diagnostic and negative results can be distressing for parents.^14^, ^15^, ^16^ ^,^ ^19^ This was highlighted by parents and HPs as a reason why post‐test support is crucial regardless of the pES result. One key difference in our study was that all parents stressed their satisfaction with having pES and found the result helpful, even if it was bad news. This may reflect the longer time that had passed since receiving results in our study, allowing parents to recover from the initial shock or disappointment of a result, compared to studies where interviews were conducted soon after return of results.^14^, ^15^, ^16^

While many HPs were concerned about potential negative impacts of reporting a VUS or IF, we could not explore this fully with the parents interviewed since none received an IF and only one couple received a VUS, which was reported with a high degree of clinical confidence. Two recent pES parent experience studies including parents who received IFs observed that parents did not always find this information useful, but the specific contribution of IFs to overall anxiety was unclear.^14^, ^16^ As experience with pES and knowledge of fetal phenotypes grows, the proportion of VUS detected will likely decrease, helping to ease HP concerns.

The complexity of the information involved has been previously noted as a challenge for supporting parents who are offered pES to make informed decisions,^20^ and our findings support previous observations that parents need time to think and opportunities to revisit key information.^17^, ^20^, ^23^ Encouragingly, the information our HPs found important to cover in pre‐test counselling corresponded closely to the informational needs expressed by parents (Table 4), and the recommendations of professional bodies.^5^ However, several parents from our (highly educated) cohort commented that written information they received about pES was difficult to understand, signalling an urgent need to develop more accessible patient information. In other areas of prenatal testing, trials of web‐based decision aids have shown promise for enabling informed decisions^28^, ^29^ but to our knowledge no such tool currently exists for pES.^30^

### 5.1 Implications for practice

This study is uniquely positioned to make recommendations for the transition of pES to safe and effective clinical implementation, based on the experience and opinion of both parents and HPs (Table 6). One recommendation was the need for national guidelines. Within the UK GMS rapid pES service, current guidance now defines clinical indications for testing and analysis is targeted to a large panel of genes associated with prenatal structural anomalies, in an effort to minimise IFs.^1^, ^11^ In line with UK guidelines for prenatal CMA,^31^ VUS detected by pES are not reported unless there is strong clinical suspicion after multidisciplinary team (MDT) discussion that the variant is relevant to the fetal phenotype. This aligns with the International Society for Prenatal Diagnosis/Society of Maternal and Fetal Medicine/Perinatal Quality Foundation joint recommendation that reporting of pES results is “best focused on pathogenic and likely pathogenic variants in genes that are relevant to the fetal phenotype”.^5^ While there remains no international consensus on broader versus more targeted analysis, an alternative approach is to perform a broader analysis but offer parents a choice of whether and when to receive any IFs.^8^, ^9^, ^10^

**TABLE 6:** Recommendations for clinical implementation of Prenatal exome sequencing (pES) made by interview participants: Health professionals (HPs) and Parents

Recommendation	Discussed by:
1. High quality, expert pre‐test counselling	Parents and HPs
Describes benefits and limitations, tailored to individual needs, simple and clear, allows parents time to think and ask questions, includes active follow‐up (see Table 4 for detailed content recommendations)	
2. Development of improved patient information resources	Parents and HPs
Standardised, accessible, written in plain language, multiple formats (e.g. web‐based, video, leaflets), multiple languages	
3. Extended support for parents	Parents and HPs
After receiving results, includes both parents, attends to emotional and psychological needs, respectful of parents' choice on ToP/continuation	
4. Clearly defined and communicated pathways and timelines for testing	Parents
for example, whether pES initiated automatically after consent or contingent on further discussion/results, in parallel with or after CMA, how and when results will be returned	
5. Education and training for HPs	HPs
Genomics education for fetal medicine professionals and fetal medicine training for genetics professionals	
6. Strengthening of multidisciplinary links	HPs
Between fetal medicine and genetics and between clinical and laboratory team members	
7. Adequate workforce and efficient pipelines	HPs
To achieve counselling, phenotyping, interpretation, laboratory technical work and maintain short turnaround with increased test uptake	
8. (Inter)National guidelines	HPs
Includes clinical indications, reporting of VUS and IFs, and addresses future reanalysis of data	

When considering MDT working, it is notable that the HPs we interviewed all worked in centres with well‐established links between fetal medicine and genetics. Wider implementation of pES may require establishing similar links. Suggestions for delivering HP training include incorporating genomics in medical and nursing school curricula, online multimedia training modules, and face‐to‐face training for consent and counselling, including role playing or clinic observation.^32^ Educational programmes should be tailored for different professional backgrounds, their self‐identified needs, and the knowledge required for their specific roles.^33^

### 5.2 Strengths and limitations

A particular strength of our study is that it combines and compares the views of parents and HPs from the same cohort. We interviewed parents with a range of experiences and outcomes, including those with and without a diagnosis from pES, those who continued or ended a pregnancy, one couple who received a VUS, and one who declined pES. In addition, HPs were recruited from centres offering rapid pES so their perspectives are largely drawn from experience. It is important to note, however, that HPs had varied levels of training or specialist interest in genomics and experience delivering pES, with some directly responsible for genetic counselling, consenting, data interpretation and delivering results, while others had more supporting roles (Table S1). The qualitative methodology used here allowed in‐depth exploration of these varied experiences and viewpoints.

An inherent limitation is that study participants were a small, self‐selected group, therefore there is a risk of responder bias in our findings. For parents there is also a risk of recall bias due to the time lag from pES offer to interview. As recruitment was conducted at centres who were early adopters of pES, the HPs we interviewed may have different views to HPs in other settings. We describe pES applied in a targeted way, intentionally limiting reporting of VUS and IFs. This reflects the approach taken in the UK GMS but means our participants had limited experience of VUS and IFs. Further, although parents had a range of experiences and outcomes, they were mostly recruited from research studies with restricted eligibility criteria and may have different perspectives from parents having pES for other reasons. As most parents offered pES had accepted, we were only able to interview one who declined and further research is required to explore parental concerns and reasons for declining. Participants were recruited from two regions in England, and almost all parents had university education, so these findings may not represent a wider population of parents and HPs. Additionally, the number of participants from each professional group is small, precluding comparisons between HPs from different training backgrounds.

## 6 CONCLUSION

We report broadly positive attitudes towards pES amongst parents and HPs with experience of pES in a research setting. Our study highlights priorities for clinical implementation, summarised in Table 6. This complex test necessitates expert counselling and interpretation, which will require further professional education and training, an increase in workforce, together with MDT working and provision for improved parental support throughout the pathway. Finally, robust consensus guidelines for referral and reporting results are required to ensure consistency and equity of access at a national level. Ongoing research as pES is implemented in clinical practice may include the generation and evaluation of parent and HP educational tools, to refine the service and ensure parent needs and preferences are met.

## CONFLICT OF INTEREST

The authors have no conflict of interest to declare.

## Supporting information

Supplementary Material

## Data Availability

The data that support the findings of this study are available from the corresponding author upon reasonable request.
