# Supplementary material for: Implementing a rapid fetal exome sequencing service: What do parents and health professionals think?
Source: Prenat Diagn. 2022 Apr 14;42(6):783–95. doi: 10.1002/pd.6140 (PMC9324936; doi:10.1002/pd.6140)
Supplement: Supplementary file 1 — Supplementary Material [file PD-42-783-s001.docx]

# Supplementary Tables

| **Characteristic** | **Professionals (N=20)** |
| --- | --- |
| **Gender**  Female  Male | 15  5 |
| **Age group (years)**  30-39  40-49  50-59  ≥60 | 6  7  6  1 |
| **Current role**  Genetic counsellor  Clinical genetics consultant  Clinical genetics trainee  Genetic scientist  Fetal medicine midwife  Fetal medicine consultant  Fetal medicine trainee | 4  3  1  2  4  4  2 |
| **Years in current role**  1-10  11-20  21-30 | 10  8  2 |

**Table S1.** Health professional characteristics

|  | **Gender** | **Age** | **Ethnicity** | **Education** | **Gestation pES offered (weeks +days)** | **ES accepted or declined** | **pES result** | **Pregnancy outcome** |
| --- | --- | --- | --- | --- | --- | --- | --- | --- |
| P1 | F | 31 | Asian/Asian British (Sri-Lankan) | Undergraduate degree | 17+5 | Accepted | No findings | Continued (Live birth) |
| P2 (partner of P1) | M | 37 | Asian/Asian British (Sri-Lankan) | Undergraduate degree |  |  |  |  |
| P5 | F | 32 | Asian/Asian British (Indian) | Not specified | 12+6 | Accepted | Diagnostic finding | ToP before pES result |
| P6 | F | 38 | White (British) | Postgraduate degree | 12+2 | Accepted | Diagnostic finding | ToP before pES result |
| P7 | F | 34 | White (British) | Undergraduate degree | 17+4 | Accepted | No findings | Continued (Live birth) |
| P8 | F | 31 | White (British) | Not specified | 21+3 | Declined | N/A | Continued (Live birth) |
| P9 | F | 44 | White (North American) | Postgraduate degree | 26+1 | Accepted | Diagnostic finding | Continued (Live birth) |
| P10 | F | 30 | White (British) | Postgraduate degree | 14+0 | Accepted | Diagnostic finding | ToP after pES result |
| P11 | F | 36 | Asian/Asian British (Indian) | Undergraduate degree | 20+5 | Accepted | Probable diagnostic finding (one LP variant and one VUS detected in trans in an AR gene) | ToP before pES result |
| P14 (Partner of P11) | M | 37 | Asian/Asian British (Indian) | Postgraduate degree |  |  |  |  |
| P12 | F | 40 | White (British) | Undergraduate degree | 21+0 | Accepted | Diagnostic finding | ToP before pES result |

**Table S2**. Parent characteristics

**Topic guides for interviews on experiences of rapid prenatal exome sequencing**

**A. For doctors, midwives, genetic counsellors**

1. Can you start by telling me about your clinical role?
2. What are your thoughts generally about using the new sequencing technologies such as WGS / WES in prenatal testing?
3. What is your experience offering WGS or WES in either a research or clinical capacity?
4. Have you ever done any training or education modules specifically about WGS or WES?
5. What do you see as the main benefits of offering rapid prenatal WES? (skeletal dysplasia panel / more widely)
   1. How is it better/different from the options for prenatal tests you have now?
   2. Can you give me some examples of the clinical benefits when you have a genetic diagnosis from WES?
6. Do you have any concerns about offering rapid prenatal WES?
7. What has been your involvement in the research study offering prenatal WES?
8. Which of your patients have you discussed rapid prenatal WES with?
   1. How is it decided who will be offered this test?
9. Can you describe the discussion you have with those patients?
   1. What do you tell them about the research study?
   2. Do you discuss the types of results such as no result or uncertain results or the limitations of the technology?
   3. What questions do parents ask?
   4. How long does that discussion tend to last?
10. What is it like for parents to make a decision about testing at that time?
    1. How long do they take to make a decision?
    2. Do you think they make an “informed decision”?
11. What can we do to better support parents? What written information do they need? Is there a place for a website or video to explain WES?
12. What are parents’ reasons for accepting the rapid prenatal WES?
13. Have you had people that decline this test?
    1. What are their reasons for declining?
14. Have you returned any results from prenatal WES to your patients?

## Can you tell me about your experience when giving results to patients?

- 1. Are comfortable discussing all possible results - Diagnosis / No primary findings / Uncertain findings

1. What are parents’ reactions to receiving results?
   1. What type of questions do parents ask?
   2. What concerns if any do they have?
2. What do you think about WES being offered during pregnancy in routine clinical practice outside a research setting?
   1. In your view, what (if any) are the main limitations of using WES for routine clinical practice?
   2. What will the main challenges be?
   3. Do you have any thoughts on who this test should be offered to or where it should sit in the clinical pathway (first line test/ after array?)
   4. Do you have any thoughts on the timing of results and decisions about termination?
3. Do you have any thoughts on what types of incidental findings, variants of uncertain significance, variable penetrance should be reported?
   1. What is it like to discuss these types of results with parents?
   2. How well do parents cope with these types of findings?
4. Specific secondary findings, such as cancer genes, were not offered in the research study, do you think there is a place for offering secondary findings in pregnancy? (for the baby? For the parents?)
   1. Do you think offering secondary findings will influence decisions about termination of pregnancy?
5. If this test is offered in clinical practice (outside research) is there anything you would like to change about how it is offered?
6. What are the key points health professionals should emphasise when offering prenatal WES?
7. Who do you think should discuss rapid WES with parents? (pre and post-testing)
   1. What is the role of genetics versus the role of fetal medicine specialists
8. What are your thoughts about how we would train health professionals that might be expected to offer WES in the future?

**B. For clinical scientists**

1. Can you start by telling me about your role?
2. What are your thoughts generally about WGS / WES?
3. Have you ever done any training or education modules specifically about WGS or WES?
4. What is your overall experience of WGS or WES in either a research or clinical capacity?
5. What do you see as the main benefits of offering rapid prenatal WES? (skeletal dysplasia panel / more widely)
   1. How does it better/different from the options for prenatal tests you have now?
   2. Can you give me some examples of the clinical benefits when you have a genetic diagnosis from WES?
6. Do you have any concerns about offering rapid prenatal WES?
7. What has been your involvement been in the research study offering prenatal WES?
8. Can you take me through the criteria for offering the test to pregnant women?
9. What are the steps you follow to accept a case and get to the point of returning a result?
10. Can you take me through the types of results that you report / don’t report and the process?
    1. What findings do you take to the MDT? Eg. Do you report everything you find and leave it to clinicians to decide what to feed back to the patient?
    2. Do you think there are any limitations with the current variant classification protocols that are used and if so what are they?
11. Can you give me some examples of the results reported as part of the study?
    1. Where there any that were VUS or no result?
12. What do you think about WES being offered during pregnancy in routine clinical practice outside a research setting?
    1. In your view, what (if any) are the main limitations of using WES for routine clinical practice?
    2. What will the main challenges be?
    3. Do you have any thoughts on who this test should be offered to? (should we be screening normal pregnancies?)
    4. Do you have any throughs on where this test should sit in the clinical pathway (first line test/ after array?)
13. Do you have any thoughts on what types of incidental findings, variants of uncertain significance, variable penetrance should be reported when the test moves to clinical practice?
14. Specific secondary findings, such as cancer genes, were not offered in the research study, do you think there is a place for offering secondary findings in pregnancy? (for the baby? For the parents?)
    1. Do you think offering secondary findings will influence decisions about termination of pregnancy?
15. What are the key points health professionals should emphasise when offering prenatal WES?
    1. What are the limitations of the test that they should point out to parents?
16. Who do you think should discuss rapid WES with parents? (pre and post-testing)
    1. What is the role of genetics versus the role of fetal medicine specialists
17. What are your thoughts about how we would train clinicians that might be expected to offer WES in the future?

**C. For parents who accepted pES**

1. Could you tell me a little background about your family and your pregnancy when you had a problem suspected following ultrasound?
2. What impact did not having a diagnosis have? What was it like to be told that your baby had a problem without knowing what the diagnosis was and what caused the problem?
   1. In terms of the information the doctors were able to provide?
   2. For you in terms of dealing with uncertainty?
3. Can you tell me about any options that were discussed with you about finding a diagnosis at this time?
4. Could you tell me what you remember about being offered whole exome sequencing as part of a research study?
   1. Who approached you?
   2. What did they discuss?
   3. Were you given any written/printed information?
   4. What is your understanding of what WES is?
   5. What were your expectations?
   6. What were you told about the results you might receive?
   7. Where any limitations of the test discussed? (prompt: such as genetic condition present that might not get picked up)
   8. What was your understanding of why you and your partner also needed to have WES?
   9. What questions did you have at the time?
   10. Did you feel you had a good understanding of WES from the information provided (written and verbal)?
5. Do you have any suggestions for how that discussion and information about WES could be improved?
   1. Where there any aspects of the discussion that were confusing or unclear?
   2. What additional information would you have liked at that time?
   3. Did you have any other support needs at this time?
6. What were the main reasons that led you to say yes to having WES?
   1. Who did you talk to when making this decision? (partner / family / health professionals)
   2. How did your partner feel about WES – where you generally in agreement?
   3. How much time did you have to make the decision? Was this enough time?
   4. What was the main reason you said yes to WES?
   5. Did you have any concerns? (miscarriage risk, data security, data sharing, insurance, timing of results and decisions about termination)
   6. In hindsight, would you make the same decision again?
7. Did you have any other types of genetic testing during your pregnancy?
8. Can you tell me about receiving the results and what that meant for you?
   1. How long did it take to get the results?
   2. When in your pregnancy did you receive the result?
   3. **[Diagnosis found]** What did the doctors tell you about condition/ genetic mutation found in the baby/ pregnancy?
   4. **[Diagnosis not found]** What did it mean for you not to have a diagnosis from this test? What did the doctors tell you at this time?
9. Do you have any suggestions for how that discussion about the results of the test could be improved?
   1. Where there any aspects of the discussion with the health professional they found confusing or unclear?
   2. What additional information would you have liked at that time?
10. Did having WES influence your decision to continue/not continue the pregnancy?
    1. What factors influenced your decision?
    2. Did having / not having the information from WES make a difference?
    3. Who did you talk to when making this decision? (partner/family / health professionals)
11. Were there particular times during the process of testing that you feel you would have benefited from more support or more information? (prompt: deciding about the test, waiting for results, deciding about termination of pregnancy)

1. How about the needs/experiences of your partner or other family members? Would they have benefited from more support or more information at any point?

General views on offering prenatal whole exome sequencing?

1. What are your overall thoughts about the test?
2. What do you think are the main benefits and disadvantages?
3. Do you have any concerns about it being offered more widely?
4. Sometimes with these sorts of tests that look across the whole genome or exome there is the option of including “incidental findings” which are gene changes not related to the main diagnosis, but could be impact on health, such as gene changes that increase your risk of cancer or heart disease. What do you think of offering incidental findings alongside WES in pregnancy – for yourself / partner / fetus?
5. What are the most important things that should be included in information sheets about prenatal WES?

**D. For parents who declined pES**

1. Could you tell me a little background about your family and your pregnancy when you had a problem suspected following ultrasound?
2. What impact did not having a diagnosis have?
   1. In terms of the way the condition was managed?
   2. In terms of the prognosis/outcome the doctors were able to provide?
   3. For you in terms of not knowing?
3. Can you tell me about any options that were discussed with you about finding a diagnosis at this time?
4. Could you tell me about your experience of being offered WES at this time?
   1. Who approached you?
   2. What did they discuss?
   3. Were you given any written/printed information?
   4. What is your understanding of what WES is?
   5. What were you told about the types of results you might receive?
   6. Were any of the limitations of the test discussed, (prompt: such as a genetic condition might be present that wasn’t picked up?; Not getting a result?)
   7. What was your understanding of why you and your partner would also need to have WES?
   8. What questions did you have?
   9. Did you feel you had a good understanding of WGS from the information provided (verbal and written)?
5. Do you have any suggestions for how that discussion and information about WES could be improved?
   1. Where there any aspects of the discussion that were confusing or unclear?
   2. What additional information would you have liked at that time?
6. What were the main reasons that led you to say no to WES?
   1. Did you have any concerns about WES? (miscarriage, data security, data sharing, insurance)
   2. Who did you talk to when making this decision? (partner / family / health professionals)
   3. How did your partner feel about having WES – where you generally in agreement?
   4. How much time did you have to make the decision? Was this enough time?
   5. Given all that was going on at this time, what was it like to be having to make a decision about WES?
   6. In hindsight, would you make the same decision again?
7. Can you tell me about how you made your decision to continue/not continue the pregnancy?
   1. What factors influenced your decision?
   2. Did not having the information from WES make a difference?
   3. Who did you talk to when making this decision? (partner/family / other health professionals)
8. Were there particular times during the process of testing that you feel you would have benefited from more support or more information? (prompt: deciding about the test, deciding about termination of pregnancy)

1. How about the needs/experiences of your partner or other family members? Would they have benefited from more support or more information at any point?

General views on offering prenatal whole exome sequencing

1. What are your overall thoughts about the test?

1. What do you think are the main benefits?
2. Do you have any concerns about it being offered more widely?
3. Sometimes with these sorts of tests that look across the whole genome or exome there is the option of including “incidental findings” which are gene changes not related to the main diagnosis, but could be impact on health, such as gene changes that increase your risk of cancer or heart disease. What do you think of offering incidental findings alongside WES in pregnancy – for yourself / partner / baby?
4. What are the most important things that should be included in information sheets about prenatal WES?
